# Bilateral repetitive transcranial magnetic stimulation ameliorated sleep disorder and hypothalamic–pituitary–adrenal axis dysfunction in subjects with major depression

**DOI:** 10.3389/fpsyt.2022.951595

**Published:** 2022-08-25

**Authors:** Xing Chen, Fei Jiang, Qun Yang, Peiyun Zhang, Haijiao Zhu, Chao Liu, Tongtong Zhang, Weijun Li, Jian Xu, Hongmei Shen

**Affiliations:** ^1^Laboratory of Biological Psychiatry, Nantong Mental Health Center & Nantong Brain Hospital, Nantong, China; ^2^Key Laboratory of Neuroregeneration of Jiangsu and Ministry of Education, Co-innovation Center of Neuroregeneration, Nantong University, Nantong, China

**Keywords:** repetitive transcranial magnetic stimulation, dorsolateral prefrontal cortex, depression, sleeping disorder, hypothalamic–pituitary–adrenal axis

## Abstract

**Objective:**

In this study, we sought to explore the effectiveness of bilateral repetitive transcranial magnetic stimulation (rTMS) over the dorsolateral prefrontal cortex (DLPFC) on depressive symptoms and dysfunction of hypothalamic–pituitary–adrenal (HPA) axis in patients with major depressive disorder (MDD).

**Materials and methods:**

One hundred and thirty-six adults with MDD were administrated drugs combined with 3 weeks of active rTMS (*n* = 68) or sham (*n* = 68) treatment. The 17-item Hamilton Depression Rating Scale for Depression (HAMD-17) was to elevate depression severity at baseline and weeks 4. To test the influence of rTMS on the HPA axis, plasma adrenocorticotropic hormone (ACTH) and serum cortisol (COR) were detected in pre- and post-treatment.

**Results:**

No statistical significance was found for the baseline of sociodemographic, characteristics of depression, and psychopharmaceutical dosages between sham and rTMS groups (*p* > 0.05). There was a significant difference in the HAMD-17 total score between the two groups at end of 4 weeks after treatment (*p* < 0.05). Compared to the sham group, the rTMS group demonstrated a more significant score reduction of HAMD-17 and sleep disorder factor (HAMD-SLD) including sleep onset latency, middle awakening, and early awakening items at end of 4-week after treatment (*p* < 0.05). Furthermore, total score reduction of HAMD-17 was correlated with a decrease in plasma ACTH, not in COR, by rTMS stimulation (*p* < 0.05).

**Conclusion:**

Bilateral rTMS for 3 weeks palliated depression *via* improvement of sleep disorder, and plasma ACTH is a predictor for the efficacy of rTMS, especially in male patients with MDD.

## Introduction

Major depressive disorder (MDD) is a disabling mental disorder characterized by depressed mood, loss of interest, and reduced drive, and it is the most prevalent, affecting approximately 15–17% of the population and showing a high suicide risk rate equivalent to around 15% (1). Recently, there have been lots of work testing the association between COVID-19 and depression in the global general populations and vulnerable subpopulations (2–5). One study suggested that the COVID-19 pandemic accelerates the increase in depressive symptoms from 19.8% in the months prior to the pandemic to 40.4% during the pandemic (6). Thus, MDD has been predicted to rank first in the total global burden of healthcare in 2030 (7).

Traditionally, pharmacological treatment and psychotherapy have been used as conventional treatment in psychiatry (8). However, one novel approach to MDD management over the last decades is repetitive transcranial magnetic stimulation (rTMS) (9). rTMS is a non-invasive brain stimulation technique for altering neuronal excitability locally by repeated magnetic pulses over the scalp (10). During the rTMS procedure, the pulsed magnetic field changes the membrane potential of neuronal cells and makes them produce an electrical current, resulting in an electrophysiological effect on the brain activity in the target area (11). Therefore, the position of rTMS coil, as well as the intensity and frequency of stimulation parameters, affect the function of rTMS on brain activity (12, 13).

In patients with MDD, functional magnetic resonance imaging (fMRI) data showed the left–right dorsolateral prefrontal cortex (DLPFC) imbalance, which is hypoactivation in the left DLPFC, and hyperactivity in the right DLPFC (14). Based on the finding, rTMS for depression typically targets the DLPFC (15). Although high (≥5 Hz) and low (≤1 Hz) frequency rTMS have opposite effects on the regional brain activity in depressed patients (13), high-frequency rTMS over the left DLPFC has therapeutic effects in MDD (16). However, high-frequency rTMS to the left DLPFC does not have effects on sleep quality in patients with depression (17). Here, we performed bilateral rTMS (combination of high and low frequency to the left and right DLPFC, respectively), which has a high quality of evidence for change in depression score (18), to examine whether the bilateral rTMS has an effect on sleep disorder in patients with MDD.

Moreover, one of the most consistent findings in the biology of depression is altered activity of the hypothalamic–pituitary–adrenal (HPA) axis (19). In depressed patients, persisting HPA system hyperactivity examined by the combined dexamethasone cortisol releasing hormone test is an important predictor for relapse and immediate maintenance therapy for rTMS (20). In addition, rats subjected to chronic unpredictable mild stress showed increased levels of adrenocorticotropic hormone (ACTH) and cortisol (COR), and rTMS reversed these changes in the treatment of depression (21). By contrast, studies also found that one session of high-frequency rTMS did not result in any mood changes, though it has a significant impact on the cortisol concentration (22, 23). Thus, the correlation between depression and levels of ACTH and COR is elusive. The aim of the current study was to test the effect of bilateral rTMS on the HPA system, especially in male patients, and examine whether the decline of concentrations of ACTH or COR is linked to the recovery of depression symptoms in patients with MDD.

## Materials and methods

The study was performed in accordance with the Declaration of Helsinki and approved by the Ethics Committee of Nantong Fourth People’s Hospital in China (Approval Number: 2018-K015), and this was a single-blinder randomized sham-controlled rTMS study.

### Subjects

One hundred and thirty-six inpatients and outpatients, independently diagnosed with MDD based on the criteria of the Diagnostic and Statistical Manual of Mental Disorders, fifth edition (DSM-V) by two psychiatrists, were successfully recruited in the study, and they were administrated antidepressants, antipsychotics, and sleep medicine normally. The dosage of these psychotropic drugs was converted according to the Guidelines for ATC classification and DDD assignment (25th edition, WHO Collaborating Centre for Drug Statistical Methods, Oslo, Norway). Briefly, the daily dosage of all kinds of psychotropic drugs in the study was found on the website,^1^ and the value of each drug was the ratio of dose (mg) in patient and daily dosage of the same drug (mg) by the website. The dosage of antidepressants, antipsychotics, and sleep medicine was the sum of the ratio of different antidepressants, antipsychotics, and sleep medicine, respectively. All participants provided written informed consent and were informed about the study procedure and the freedom to leave the study for any reason. All procedures were performed in Nantong Fourth People’s Hospital from October 1, 2018 to December 31, 2019.

Exclusion criteria included: (1) severe physical diseases, (2) alcohol or drug abuse, (10) epilepsy, (4) brain injury, (5) stroke, (6) pregnant and lactating women, and (7) inability to understand instructions.

### Repetitive transcranial magnetic stimulation and sham intervention

The participants were randomly assigned to two types of interventions (active rTMS and sham). rTMS stimulation was delivered using the transcranial magnetic stimulator (CCY-I, Yiruide Co., Ltd., Wuhan, China) with a circular coil at a diameter of 126 mm. Prior to the first rTMS treatment, the TMS intensity for each patient was determined based on the resting motor threshold (rMT), which evokes the motor potential in the abductor polis brevis muscle. According to rTMS studies for depression, stimulation was applied in bilateral DLPFC, 5 cm anterior to the scalp position for the motor potential. In the rTMS group, 68 patients completed 15 sessions of TMS over bilateral DLPFC, administrated daily, 5 days per week, for a 3-week period. One thousand one hundred fifty pulses of 10 Hz excitatory TMS were applied over the left DLPFC by 5-s trains with a 35-s inter-train interval, and then 600 pulses of 1 Hz inhibitory TMS were delivered over the right DLPFC *via* 10-s trains with a 5-s inter-train interval. Sham treatment was applied using the same procedure as the TMS treatment, but the TMS coil was positioned at a reversed outward by 90° angle to the rTMS real stimulation.

### Hamilton depression rating scale

The level of depression was assessed by the 17-item Hamilton Depression Rating Scale (HAMD-17), and higher HAMD-17 scores indicated more severe depression. HAMD-17 contains five factors, including anxiety/somatization (HAMD-A/S), retardation (HAMD-R), cognitive disorder (HAMD-CD), sleep disorder (HAMD-SLD), and weight (HAMD-W). In addition, HAMD-SLD includes items of sleep onset latency, middle awakening, and early awakening. In the study, the depression symptoms of patients with MDD were tested at baseline prior to the first rTMS treatment and at the end of 4 weeks after rTMS treatment by HAMD-17.

### Adrenocorticotropic hormone and cortisol measurement

Three milliliters of peripheral blood were taken from the antecubital vein of each subject pre- and post-treatment at 06:30 a.m., and the plasma and the serum were obtained after centrifuging at 3500 rpm for 10 min. Then, the samples were collected and preserved at −80°C until analysis. Levels of plasma ACTH and serum COR were measured by anti-ACTH and anti-COR chemiluminescent microparticle immunoassay kits, respectively (Autobio diagnostics Co., Ltd., Zhengzhou, China). The assay is based upon the two-step sandwich method. In the first step, the samples were added to the microparticles for ACTH and COR binding to the antibodies. In the second step, the microparticles, which captured ACTH or COR, were allowed to react with enzyme-linked antibodies. After that, the chemiluminescent substrate was added and catalyzed by the complex among the microparticles, resulting in a chemiluminescent immunological reaction, which was measured by Autolumo A2000 Plus.

### Statistical analyses

All statistical analyses were conducted with the SigmaPlot 13.0 and IBM SPSS Statistics 26 software. *Shapiro–Wilk* and *Brown–Forsythe* test were used to ensure that the data were normality and equal variance, respectively. For the comparison of the two groups, data were analyzed by the independent *Student’s t*-test. One-way ANOVA or two-way ANOVA was used to examine the difference among more than two groups, and *Student–Newman–Keuls* method was performed to analyze the pairwise multiple comparisons to identify significance between different groups. The data that did not pass the normality and equal variance tests were used *Mann–Whitney Rank Sum* test for the comparison of two groups, and *Kruskal–Wallis* one-way analysis of variance on ranks for the difference among more than two groups. *Holm–Sidak* method was used for *post-hoc* analysis to identify significantly different groups. Finally, relevancy was examined using *Pearson’s Product Moment* correlation. All statistical analysis was two-tailed and *p* < 0.05 was considered significant. The specific statistical analysis method for each experiment is described in Table footnote. Data are reported as means ± SEM.

## Results

### Demographic and baseline information of repetitive transcranial magnetic stimulation and sham groups

**FIGURE 1:**
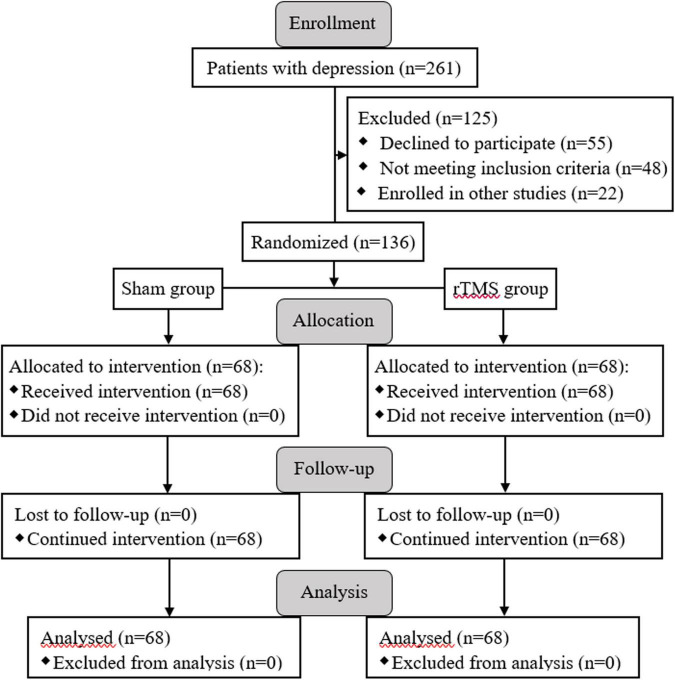
Enrollment, allocation of groups, and follow-up processes are presented with the flow diagram.

A total of 136 subjects with major depression was successfully recruited for the final data set, and they were randomly divided into two groups: rTMS group (*n* = 68) and sham group (*n* = 68) (see Figure 1). There was no significant difference between the two groups in socio-demographic data, including gender, age, marital status, and education (*p* > 0.05, see Table 1). At the beginning of the study, we compared the characteristics of baseline between the rTMS stimulation group and sham group to check the homogeneity of their performance in the family history of psychiatric disorders, duration of illness, first-episode, psychiatric inpatient care, dosage of psychotropic drugs, and the scores of HAMD-17 before administering intervention. There was no significant difference in the pre-test features between the groups (*p* > 0.05, see Table 1).

**TABLE 1:** Demographic and baseline characteristics of sham and repetitive transcranial magnetic stimulation (rTMS) groups.

Variable	Sham group (*n* = 68)	rTMS group (*n* = 68)	*t*/*x*^2^	*p*
Gender (male/female)	35/33	36/32	0.029	0.864^a^
Age (years)	47.265 ± 1.729	50.221 ± 1.820	1.177	0.241
Marital status (single/married/widowed)	6/58/4	7/60/1	4.134	0.247^a^
Education (years)	8.058 ± 0.246	7.971 ± 0.284	0.235	0.815
Family history (yes/no)	4/64	5/63	0.119	0.730^a^
Duration of illness (months)	44.132 ± 3.048	37.206 ± 3.625	1.462	0.146
First-episode (yes/no)	27/41	24/44	0.282	0.595
Inpatient care (yes/no)	20/48	29/39	2.584	0.108^a^
Antidepressants dosage	1.202 ± 0.0779	1.283 ± 0.0970	0.63	0.52
Kinds of antidepressants (SSRI/SNRI/others)	19/33/16	28/27/13	2.634	0.268^a^
Antipsychotic dosage	0.430 ± 0.0456	0.385 ± 0.0394	0.74	0.461
Sleeping dosage	0.496 ± 0.0874	0.528 ± 0.0961	0.182	0.856
Total score of HAMD-17	30.897 ± 0.740	30.809 ± 1.003	0.071	0.944
HAMD-A/S	9.206 ± 0.280	8.853 ± 0.469	0.646	0.52
HAMD-W	0.603 ± 0.0598	0.618 ± 0.0785	0.149	0.882
HAMD-CD	7.632 ± 0.207	7.191 ± 0.279	1.269	0.207
HAMD-R	9.397 ± 0.237	9.397 ± 0.250	0.000	1.000
HAMD-SD	4.559 ± 0.131	4.750 ± 0.143	0.985	0.326

Values are presented as mean ± SEM.

^a^Chi-square analysis.

rTMS, repetitive transcranial magnetic stimulation; SSRI, selective serotonin reuptake inhibitor; SNRI, serotonin and norepinephrine reuptake inhibitor; HAMD-17, 17 items Hamilton depression rating scale; HAMD-A/S, HAMD-anxiety/somatization factor; HAMD-W, HAMD-weight factor; HAMD-CD, HAMD-cognitive disorder factor; HAMD-R, HAMD-retardation factor; HAMD-SLD, HAMD-sleep disorder factor.

### Improvement of repetitive transcranial magnetic stimulation on depressive symptoms

**FIGURE 2:**
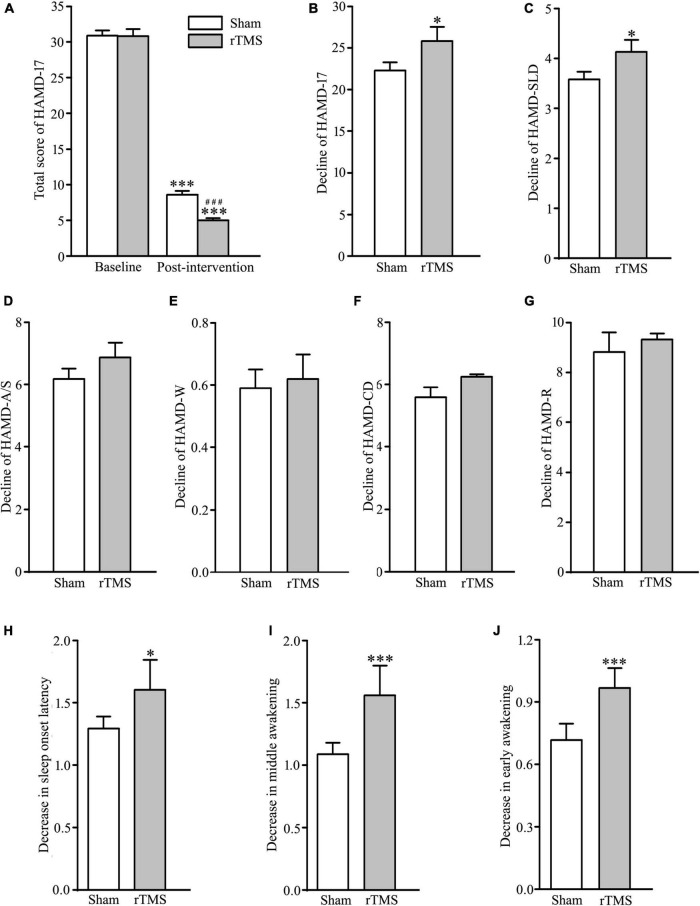
Effect of repetitive transcranial magnetic stimulation (rTMS) on depression scores measured with 17 items Hamilton depression rating scale (HAMD-17) at baseline and 4-week after bilateral rTMS or sham treatment in patients with major depressive disorder (MDD) combined with psychotropic drugs. **(A)** Total score of HAMD-17 at baseline and 4 weeks after treatment. **(B)** Change in a total score of HAMD-17 from baseline to post-intervention at 4 weeks. **(C–G)** Change in scores of five factors in HAMD-17; HAMD-SLD **(C)**, HAMD-A/S **(D)**, HAMD-W **(E)**, HAMD-CD **(F)**, and HAMD-R **(G)**. **(H–J)** Change in scores of three items in HAMD-SLD; sleep onset latency **(H)**, middle awakening **(I)**, and early awakening **(J)**. rTMS group significantly different from the sham group was marked by asterisks. ^***^p < 0.001, compared with the baseline **(A)**; ^###^*p* < 0.001, compared with the sham group at 4 weeks after treatment; *p < 0.05, ^***^p < 0.001, compared with the sham group **(B–J)**. Graphs show mean ± SEM; *n* = 68.

In order to assess the intervention of rTMS on depressive symptoms, we performed bilateral rTMS (combination of high and low frequency to the left and right DLPFC, respectively), which has a high quality of evidence for change in depression score (18). The results of two-way ANOVA analysis have shown that HAMD-17 total scores, indicating subjective depressive symptoms, significantly decreased in both sham stimulation and rTMS groups as treatment progressed, *F* = 1172.507, *DF* = 1, *p* < 0.001, and the HAMD-17 total scores at 4 weeks after intervention were significantly deferent between sham and rTMS groups, *F* = 6.904, *DF* = 1, *p* = 0.009. However, *Holm–Sidak* multiple comparisons analyses showed no difference between sham and rTMS groups in baseline (*t* = 0.0888, *p* = 0.929), while other comparisons (baseline vs. 4 weeks after treatment, *t* = 22.444, *p* < 0.001 in the sham group, and *t* = 25.982, *p* < 0.001 in the rTMS group; sham *vs*. rTMS at 4 weeks after treatment, *t* = 3.627, *p* < 0.001) displayed statistical significance (see Figure 2A). In addition, we also computed *Student’s t*-test to verify the validity of rTMS stimulation on major depression by the decline in the HAMD-17 total scores from baseline to post-intervention at 4 weeks. We found a statistically significance between rTMS and sham groups (rTMS *vs.* sham group, 25.809 ± 1.025 vs. 22.294 ± 0.976, *t* = 2.482, *p* = 0.0143; see Figure 2B).

The 17 items in HAMD-17 were separated into five factors: HAMD-A/S, HAMD-W, HAMD-CD, HAMD-R, and HAMD-SLD (24). Out of the five factors, *t*-test analysis showed that HAMD-SLD score of the experimental group was decreased significantly following rTMS intervention (rTMS vs. sham group: 4.132 ± 0.151 vs. 3.588 ± 0.149, *t* = 2.570, *p* = 0.0112; see Figure 2C), while other factors did not reach statistical significance (rTMS vs. sham group: 6.868 ± 0.474 vs. 6.147 ± 0.333, *t* = 1.244, *p* = 0.216, for HAMD-A/S; 0.618 ± 0.0785 vs. 0.588 ± 0.0601, *t* = 0.298, *p* = 0.767 for HAMD-W; 6.353 ± 0.296 vs. 5.897 ± 0.282, *t* = 1.115, *p* = 0.267 for HAMD-CD; 9.324 ± 0.238 vs. 8.823 ± 0.279, *t* = 1.363, *p* = 0.175 for HAMD-R; see Figures 2D–G). Therefore, the antidepressant efficacy of rTMS stimulation in patients with major depression was achieved by improving sleep quality. It is well known that HAMD-SLD includes item 4 (sleep onset latency), 5 (middle awakening), and 6 (early awakening) (25), and an independent sample *t*-test was conducted to compare the decline of the three sleep items from baseline to post-intervention at 4 weeks. The results showed that the improvement of sleep quality by rTMS stimulation caused by reducing sleep items 4, 5, and 6, especially in items 5 and 6 (rTMS vs. sham group: 1.603 ± 0.0842 vs. 1.294 ± 0.0962, *t* = 2.417, *p* = 0.0170, for item 4; 1.559 ± 0.0822 vs. 1.088 ± 0.0907, *t* = 3.845, *p* < 0.001 for item 5; 1.529 ± 0.0678 vs. 0.720 ± 0.0779, *t* = 7.829, *p* < 0.001 for item 6; see Figures 2H–J). Taken together, rTMS stimulation has antidepressant efficacy by the reduction of sleep disturbances.

### Improvement of repetitive transcranial magnetic stimulation on hypothalamic–pituitary–adrenal dysfunction

**FIGURE 3:**
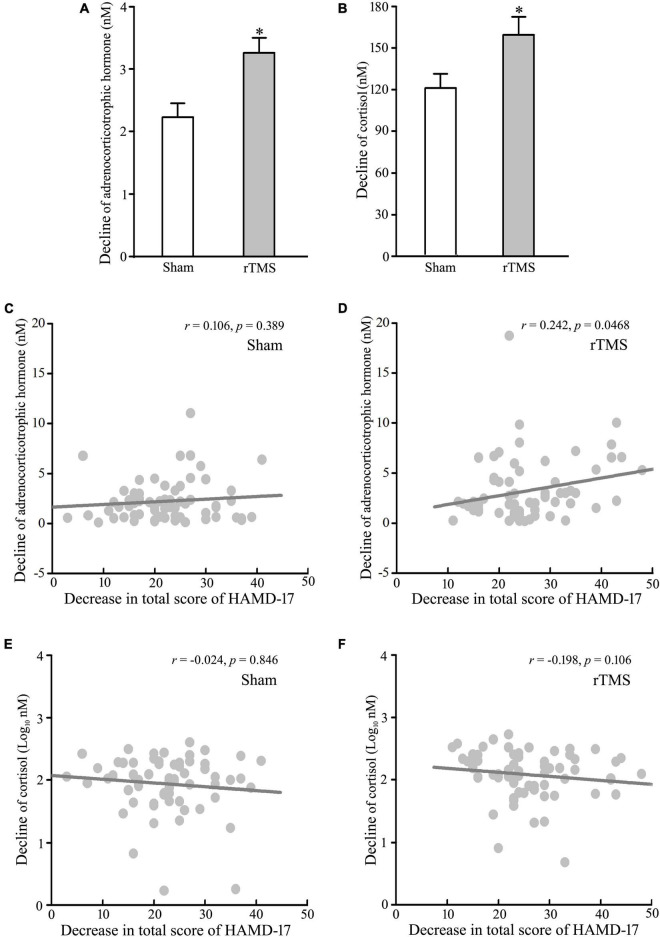
Effect of repetitive transcranial magnetic stimulation (rTMS) on the hypothalamic–pituitary–adrenal (HPA) axis. Plasma adrenocorticotropic hormone (ACTH) and serum cortisol (COR) were detected at baseline and 4-week after rTMS or sham treatment. **(A,B)** Change in the concentration of ACTH **(A)** and COR **(B)** from baseline to post-intervention at 4 weeks. **(C,D)** Correlation analysis of change in ACTH to decline in a total score of 17 items Hamilton depression rating scale (HAMD-17) in the sham group **(C)** and rTMS group **(D)**. **(E–F)** Correlation of change in COR to decline in a total score of HAMD-17 in the sham group **(E)** and rTMS group **(F)**. rTMS group significantly different from the sham group was marked by asterisks. *p < 0.05, compared with the sham group **(A,B)**. Graphs show mean ± SEM; *n* = 68.

The abnormal upregulation of HPA is one of the most robust pathophysiologies of major depressive disorder, and novel approaches, which target the pathophysiological feature, have antidepressant effects (19, 26). Therefore, we tested the decline of the blood levels of ACTH and COR from baseline to post-intervention at 4 weeks. The analysis of *t*-test showed that the levels of ACTH and COR were decreased significantly following rTMS stimulation (rTMS *vs*. sham group: 3.258 ± 0.374 vs. 2.232 ± 0.220, *Mann–Whitney U* Statistic = 1843.000, *p* = 0.041, for ACTH, see Figure 3A; 159.384 ± 13.143 vs. 121.188 ± 10.121, *Mann–Whitney U* Statistic = 1797.500, *p* = 0.025, for COR, see Figure 3B). Then, the correlation between the reduction of HPA axis function and the antidepressant effect of rTMS stimulation was tested by *Pearson’s* analysis. The results revealed that the decline of the plasma ACTH level, not the serum COR level, was significantly positively correlated with the reduction of HAMD-17 total score only in the rTMS group (*r* = 0.106, *p* = 0.389, for ACTH in the sham group; *r* = 0.242, *p* = 0.046, for ACTH in the rTMS group; *r* = −0.0240, *p* = 0.846, for COR in the sham group; *r* = −0.198, *p* = 0.106 for COR in the rTMS group, see Figures 3C–F). Thus, ACTH might play an important role in the amelioration of rTMS on depressive symptoms. Taken together, our results suggest that bilateral rTMS over DLPFC improved sleep quality, and ACTH, rather than COR, was associated with the efficacy of bilateral rTMS.

### Gender-specificity of repetitive transcranial magnetic stimulation on depressive symptom

**FIGURE 4:**
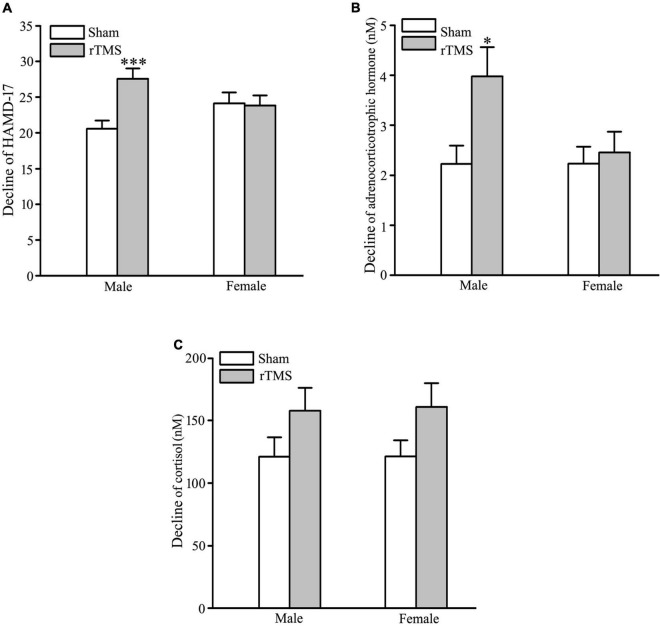
Gender-specificity of repetitive transcranial magnetic stimulation (rTMS) on depressive symptom and hypothalamic–pituitary–adrenal (HPA) axis. **(A)** Change in the total score of 17 items Hamilton depression rating scale (HAMD-17) from baseline to post-intervention at 4 weeks in male and female patients. **(B,C)** Change in the concentration of adrenocorticotropic hormone (ACTH) **(B)** and cortisol (COR) **(C)** from baseline to post-intervention at 4 weeks in male and female patients. rTMS group significantly different from the sham group was marked by asterisks. *p < 0.05, ^***^p < 0.001, compared with the sham group. Graphs show mean ± SEM; *n* = 68.

In order to test the gender-related differential response to rTMS stimulation, sham and rTMS groups were divided into male and female subgroups (male vs. female: 35 vs. 33 in the sham group; 36 vs. 32 in the rTMS group). The results of ANOVA analysis have shown that there was a significant difference in the decline of HAMD-17 total scores from the baseline to post-intervention at 4 weeks among the four subgroups, *F* = 4.434, *DF* = 3, *p* = 0.005 (see Figure 4A). *Student–Newman–Keuls* multiple comparisons analyses have revealed that the decrease of HAMD-17 total score in the rTMS group was significantly higher than in the sham group among male patients (*q* = 5.151, *p* = 0.002), while other comparisons did not reach the statistical difference (see Figure 4A). We also analyze the difference in the decline of ACTH and COR from baseline to post-intervention among the four subgroups. The results of ANOVA analysis have shown that there was a statistically significant difference in the decline of ACTH, not in the decrease of COR, among the treatment groups, *F* = 3.791, *DF* = 3, *p* = 0.012, in ACTH; *F* = 1.747, *DF* = 3, *p* = 0.161 (see Figures 4B,C). Multiple comparisons analyses have revealed that the decrease of ACTH in the rTMS group was significantly greater than in the sham group among male patients (*q* = 4.051, *p* = 0.022), while other comparisons did not reach the statistical difference (see Figures 4B,C). Therefore, the antidepressant efficacy of rTMS stimulation is sex specificity.

### Adverse effects

In the sham group, two cases reported mild nausea after 1 week, and three cases had dry mouth and constipation 3 weeks after treatment. Therefore, five cases (7.353%) reported at least one adverse effect during the course of treatment in the sham group. In the rTMS group, total seven cases (10.294%) reported one adverse effect, of which four patients complained of mild headache at the first rTMS treatment, and three cases reported dry mouth and constipation, as the patients in the sham group experienced. No significant differences were identified between the two groups.

## Discussion

The results of this study showed that bilateral rTMS over DLPFC improved sleep quality, and facilitated the reversal of ACTH and COR levels in patients with MDD. However, ACTH, rather than COR, was linked to the efficacy of bilateral rTMS on depression. Therefore, our study provided a new protocol of rTMS to MDD management and indicated that ACTH is a biological predictor of depression state.

Repetitive transcranial magnetic stimulation is a non-invasive brain stimulation technique for clinical treatment (27), and the original protocol for rTMS, which was approved by the United States Food and Drug Administration in routine clinical practice as a treatment for MDD, is the 10 Hz left DLPFC, and it involves one session in each working day and patients need to come for the treatment for continuously 4 weeks (28). Recently, fMRI data showed the hypoactivation in left DLPFC and hyperactivity in the right DLPFC in patients with MDD (14). In addition, high-frequency TMS stimulation induces the potentiation of neuronal activity, while low-frequency stimulation evokes depressive function on neuronal activity, thus mimicking neuroplasticity through long-term potentiation and long-term depression, respectively (29). Recently, clinical practice changes from unilateral to bilateral rTMS treatment or modifies the model of rTMS stimulation from high-frequency left DLPFC to low-frequency right DLPFC (30). In this clinical study, we combined high-frequency left DLPFC and low-frequency right DLPFC to examine the efficacy of rTMS treatment on depression. Due to a delayed response to rTMS (31), patients with MDD were treated continuously for 3 weeks, then waited for 1 week for the measurement of depression to elevate the clinical improvement from the treatment.

Hamilton Depression Scale is the most commonly used scale in the clinical evaluation of depression, and the scale has three versions: 17 items, 21 items, and 24 items (32). Among the three versions, 17-items HAMD includes HAMD-A/S, HAMD-W, HAMD-CD, HAMD-R, and HAMD-SLD. In the study, the total score of 17-items HAMD and the score of its five factors were assessed pre- and post-treatment. At baseline, these scores were no difference between sham and rTMS groups (Table 1), and the total score of HAMD-17 decreased significantly in sham and rTMS groups, comparison with the baseline, at end of 4 weeks after treatment because of administrated drugs (Figure 2A). In addition, there was a significant difference between the sham and rTMS groups in the total score of HAMD-17 at 4 weeks after treatment (Figure 2A). This suggested that rTMS treatment can promote the recovery of depression. To confirm the effect of rTMS stimulation on depression, we compared the decline from baseline to the end of 4 weeks after treatment between sham and rTMS groups in the scores of HAMD-17 and its five factors (Figures 2B–J). The results verified that rTMS intervention had an influence on the recovery of depression based on the decline of the total score of HAMD-17 (Figure 2B). Furthermore, rTMS stimulation improved sleep quality in patients with MDD, namely, initiating sleep difficulty, shallow sleeping, and early awaking (Figures 2G–J). Thus, 3-week course of bilateral rTMS over DLPFC confers the improvement of mood and sleep quality. In the previous studies, 20 daily sessions of 10 Hz rTMS over the left DLPFC does not have effects on sleep quality in patients with depression (17), while the course of the unilateral rTMS, which can improve depression and sleep measured by Pittsburgh Sleep Quality Index Patient Health Questionnaire-9, is 6 weeks (33). Therefore, the effectiveness of rTMS in treating depression changes from unilateral to bilateral (28), and 1 Hz rTMS over the right DLPFC can accelerate the recovery of depression and sleep disorder.

Since the 1960s, the influence of HPA axis activity on the pathophysiology of depression has been extensively studied (34). Up to date, it is not clear if the dysregulation of the HPA axis actually caused depression or if some other feature of depression is responsible for HPA malfunction (35). However, some depressive symptoms result from the dysfunction of the HPA axis (36), and elevated ACTH and COR levels have been repeatedly reported in depression patients (37, 38). Thus, we collected the blood specimens from the patients with MDD during pre- and post-treatment to test the effect of rTMS stimulation on ACTH and COR levels. Consistent with the data of HAMD-17 total scores, the results showed that the concentrations of plasma ACTH and serum COR decreased after 4 weeks of treatment in sham and rTMS groups (Figures 3A,B), though neither acute nor chronic rTMS affected basal ACTH and COR levels in male Albino rats (39, 40). In addition, the decreased degree of ACTH and COR levels in the rTMS treatment group was obviously higher than those of the sham group (Figures 3A,B). Thus, the decline of concentrations of ACTH and COR might be linked to the recovery of depression symptoms in patients with MDD. In order to confirm the hypothesis, we analyzed the correlations between lower measurements of the total scores of HAMD-17 and decreased levels of ACTH and COR in sham and rTMS groups. Interestingly, there was no association of a decrease in the COR level with a decline of total scores of HAMD-17 in sham and rTMS groups, but a decrease in ACTH was correlated with a decline of total scores of HAMD-17 only in the rTMS group (Figures 3C,D). Therefore, ACTH, rather than COR, can be linked to the effect of bilateral rTMS on depression in patients with MDD.

In healthy female volunteers, previous study reported that an immediate or delayed impact of one session of high-frequency rTMS on the left or the right DLPFC on the salivary COR level did not find (41–43). In this study, we further examined whether bilateral rTMS stimulation had an effect on the HPA axis, by analyzing plasma ACTH and serum COR levels in male and female patients with MDD. In female patients with MDD, rTMS stimulation produced less depression and HPA activity across 4 weeks of treatment, but neither differed from the sham intervention (Figures 4A–C). Contrary to the findings in female patients with MDD (43), our results manifested that rTMS did promote a less depressive state and plasma ACTH concentration of male patients with MDD, relative to the sham group (Figures 4A,B). For serum COR level, our data did not indicate that there was a significant difference between sham and rTMS stimulation in male MDD participants (Figure 4C). Recently, fMRI findings have revealed that male patients with MDD exhibited increased neural stress responses in the DLPFC and frontoparietal network (PFN) relative to females, and less deactivation in limbic-striatal regions, namely, the amygdala, hippocampus, and nucleus accumbens (NAc) was only observed in female patients with MDD (44). Therefore, the effect of bilateral rTMS over DLPFC on depression and HPA function was only observed in male patients with MDD. Our findings confirm the importance of considering gender-difference when developing a novel method for MDD treatment. Collectively, these results indicated that ACTH, rather than COR, might serve as a biological predictor of the effect of rTMS stimulation on depression, especially in male patients with MDD.

In summary, our data indicated that bilateral rTMS ameliorated depression *via* the improvement of sleep disorders in MDD, and ACTH concentration in blood is a biomarker to predict the efficacy of rTMS in male patients with MDD. In future, we will explore the mechanism of the association between ACTH and rTMS stimulation.

## Data availability statement

The raw data supporting the conclusions of this article will be made available by the authors, without undue reservation.

## Ethics statement

The studies involving human participants were reviewed and approved by Ethics Committee of Nantong Fourth People’s Hospital. The patients/participants provided their written informed consent to participate in this study.

## Author contributions

XC, JX, and HS contributed to the conception and design of the study. FJ, QY, CL, PZ, TZ, and HZ collected the data. XC and WL organized the database. XC and HS performed the statistical analysis. HS wrote the manuscript. All authors contributed to the article and approved the submitted version.

## Footnotes

1
www.whocc.no
